# Cognitive output of a neuropsychological stimulation program in an elderly day care center with low educated participants: An observational study

**DOI:** 10.1590/S1980-57642014DN82000012

**Published:** 2014

**Authors:** Maria Vânia Silva Nunes, Ana Almeida Pinho, Helena Mauricio Campos, Paula Abreu, Isabel Pinto Gonçalves, Alexandre Castro Caldas

**Affiliations:** 1 The Catholic University of Portugal. Health Sciences Institute.; 2 Center for Interdisciplinary Research in Health (CIIS).; 3 Lisbon Branch of Portuguese Red Cross.

**Keywords:** elderly, low education, community, neuropsychological stimulation, dementia

## Abstract

**Objective:**

In the present paper we present an observational study of the implementation of a Neuropsychological Stimulation Program at an Elderly Day Care Center in low-educated participants with very similar backgrounds concerning social economic status.

**Methods:**

The implemented program tackled several dimensions, including daily orientation sessions, cognitive stimulation sessions twice a week, followed by movement sessions, and structured sessions conducted every two weeks. Cognitive Evaluation was performed before and after implementation of the program.

**Results:**

Results are discussed taking into consideration cognitive outputs as well as non-cognitive outputs and the specificities of community-based intervention.

**Conclusion:**

It was concluded that community-based intervention is set to become vital in promoting dementia prevention.

## Abstract (pt)

**Objetivo:**

No presente artigo, apresentamos um estudo observacional sobre a implementação de um Programa de Estimulação Neuropsicológica em um Centro Dia para idosos, envolvendo idosos de baixa escolaridade com perfil socioeconômico homogêneo.

**Métodos:**

O programa implementado abordou várias dimensões, incluindo sessões diárias de orientação, sessões de estimulação cognitiva duas vezes por semana, seguidas de sessões de atividade motora, e sessões estruturadas que foram conduzidas a cada duas semanas. A avaliação cognitiva foi conduzida antes e depois da implementação do programa.

**Resultados:**

Os resultados são discutidos levando em consideração desfechos cognitivos e não-cognitivos e as especificidades da intervenção de base comunitária.

**Conclusão:**

Concluiu-se que a intervenção com base na comunidade será vital para a prevenção das demências.

## INTRODUCTION

Portugal is presently the 6º most-aged country in the world. Although an ageing population is common to Europe as a whole, the factor distinguishing the Portuguese case is the speed at which the aging process has occurred.^1^ In fact, at the present, the fecundity index in Portugal is extremely low.^2^ The ageing process and its speed in the Portuguese case places additional demands on health and social systems. Although there is now a clear focus on active ageing and promoting quality of life during the entire life cycle, at least at national^3^ and European policy levels, specifically at the level of life-long learning, its translation to intervention and action is not always clear.^4^ Nevertheless, it is a consensus that both health and social systems should promote active lives throughout the whole life cycle, considering a multidimensional approach to the individual.

Despite several advances in the integration of the Portuguese health and social systems in recent years, the change in disease pattern has outstripped the ability of the system to translate from a more acute approach to an integrated approach capable of fully dealing with the changes in the disease pattern, namely, the increase in non-communicable diseases, including dementia. This has led to an increased level of deterioration, at physical, cognitive and functional levels, of the users of care services that are eminently social in nature i.e. elderly day care centers. In Portugal, akin to other countries, elderly day care centers are social entities seeking to promote the maintenance of the elderly in their socio-familial environment through the provision of basic needs, psycho-social support, and occupation, thereby preventing isolation.^5^ While constituting the social service solution with least dependence, mainly due to its community-based nature, and as compared to residential entities,^6^ there is nevertheless a recognized trend that the dependence level is increasing concomitantly with an increase in the age of the users.

There has been an increase over the time in the educational level of the elderly Portuguese population. Nevertheless, in 2013, 31.4% of the resident population over 65 years of age had no schooling, 47.3% had 0 to 4 years, 3.7% 5 to 6 years, 7.8% 6 to 9 years, 3.6% 10 to 12 years, while only 6.2% had education at university level.^2^ At the same time, elderly day care centers are still associated with relatively low-income users commensurately increasing the percentage of users with lower educational levels.

Both higher age and low educational levels are factors associated with increased risk for cognitive decline. The effect of education on age-related cognitive decline is complex. As pointed out in an early study,^7^ the educational effect is not linear, representing a kind of "negatively accelerated curve". This means that at lower levels of education, differences between 0 and 3 years of formal schooling are highly significant, whereas differences between 12 and 15 year of education are irrelevant. The authors also demonstrated that different patterns of association could be expected for age-related decline and education.^8^ Nevertheless, it seems a general consensus that education has a positive effect on cognitive status.

Several mechanisms have been proposed to explain how educational attainment can be protective against cognitive decline, such as the "use it or lose it" perspective^9^ or the cognitive reserve hypothesis.^10^

Cognitive reserve is a term that was coined^11^ by Stern (2002) in an attempt to account for the disjunction between the degree of brain pathology and functional manifestation of the pathology or lesion. This construct aims to explain how individual differences in neural networks or cognitive processing may underlie different ways of coping with brain pathology or lesion.

In a review of the concept based on epidemiological evidence, Stern pointed to measures of socioeconomic status (SES), as proxies for cognitive reserve, such as income or occupational attainment, educational attainment and leisure activities. According to this perspective, education, income and occupational attainment are protective factors against several brain disorders, including dementia.^12^ It is widely accepted that the risk of dementia and Alzheimer disease in old age is reduced in persons with higher levels of educational attainment compared to those with lower levels. The concept of Cognitive Reserve is very influential and corroborated by animal models.^13^

Surprisingly, longitudinal results from the Victoria Longitudinal showed that although education is linked to cognitive performance it was found to be unrelated to cognitive decline.^14^ These results are supportive of a passive reserve hypothesis, where individuals with higher school attainment are always better but decline at the same rate. Even under this more passive perspective, education is protective since more pathology can be endured until the deficits become clear.

Kramer et al. (2004),^15^ in a review examining the evidence for and against the "use it lose it" perspective, noted that education is not the only measure of environmental and experiential richness nor is it a pure measure of the construct. As expected, they found that support from cross-sectional studies for the beneficial effect of education in the aged is considerable. Nevertheless, as observed in longitudinal studies, the influence of education was not uniform with respect to cognition. As highlighted by the authors, the research reviewed suggests unequivocally that education can serve as an effective moderator of cognitive vitality in late adulthood but that its interaction with other factors such as lifestyle, SES and occupational complexity has yet to be clearly established. This may be why results regarding the impact of education on the rate of cognitive decline are not clear, especially when considering that heterogeneity increases with age.^16^

Nevertheless, considering the proxy measures of cognitive reserve outlined by Stern^12^ it is expected that elderly persons with low educational level, low socio-economic status and undemanding occupational activities have less cognitive reserve, being more vulnerable to the effects of brain pathology. These are the characteristics of the majority of elderly persons currently attending elderly day care centers. Although aging is not a lesion in a neurological sense, it is associated with neurological deterioration, reinforcing the notion that preventive strategies have to be implemented in order to try to minimize these risks.

As noted by Lachman et al.^17^ "if participating in accessible cognitive activities can compensate for a significant portion of the cognitive disadvantage of lower education in adulthood, there is a promise for modifying risks of cognitive decline in less advantaged population, by encouraging frequent engagement in relatively accessible activities". Despite this, intentional interventions in a community-based context are still relatively sparse.

Therefore, the aim of the present work was to implement and evaluate a neuropsychological stimulation program in a community-based setting, namely an elderly day care center, with low-educated participants, as part of a prevention dementia program. The observational study conducted is presented below.

## METHOD

**Participants.** All the participants were recruited in an elderly day care center where the program was run with the collaboration of those responsible and staff. The subjects were all Portuguese native speakers. Participants were screened for substance abuse, psychiatric or neurological problems, and developmental disorders by means of a structured interview. From an original sample of 22 participants initially enrolled, 10 were excluded from the analysis. The reasons for exclusion were mainly due to neurological (including dementia) and psychiatric diseases; one of the participants was excluded due to her relatively high number of years of schooling. None of the included participants fulfilled the criteria for dementia according to the DSM-IV, and all maintained social functioning by attending the elderly day care center.

The final sample size was 12 participants, comprising four male and eight female participants with mean schooling of 4.07±0.267 years and age of 73.29±10.78 years.

Although small, the sample had the advantage of being homogeneous not only in terms of years of schooling, but also for professional occupation and social and relationship network.

A relevant aspect of the sample was that the elderly had very similar social experiences and similar interactions during their entire life course. They all lived in the same social environment (same neighborhood), shared similar incomes (according to social evaluation), as well as the same occupations (all the women were housewives or domestic services employees and all the men were blue-collar workers). It is often pointed out that social economic status (SES) should be taken into consideration as a factor associated with low education, as well as other aspects, such as social interactions, but it is often hard to find a population homogeneous on all factors, thereby allowing a similar cognitive reserve to be assumed.

The elderly day care center is placed within and serves a very small "economical" neighborhood where, in the late 1940s, reproducing an idyllic image of a "country village", one or two family houses were built with a small backyard and land plot dedicated to growing their own vegetables in peripheral locations relative to the central areas of the city,^18^ which include one church, school and sport facilities. Participants have lived their lives in this same neighborhood, allowing a great similarity in their educational, social, relational and leisure backgrounds for a long period of time.

**Procedure.** Two neuropsychologists, a clinical psychologist and a movement technician under the supervision of a neuropsychology professor applied the program for about 10 months. Other members of the staff were also enrolled.

As pointed out previously, aging is not a lesion in a neurological sense, but is known to be associated with neurological deterioration which in turn has cognitive implications, among others. Therefore, although this was a stimulation rather than a rehabilitation program, some of the premises involved in cognitive rehabilitation were used,^19^ to accommodate the notion that addressing only cognition would be insufficient, and that emotion and psychosocial functioning are interlinked and should all be targeted in the best possible way. It is also recognized that cognitive rehabilitation should also address associated problems such as mood or behavioral problems. The importance of working together was also taken into consideration and different members of the staff were involved in the activities plan, attending regular meetings.

The program consisted of interventions involving different dimensions including daily orientation sessions, cognitive stimulation sessions twice a week, followed by movement sessions, and structured sessions that were conducted every two weeks. The structured sessions consisted of 13 loosely adapted cognitive stimulation sessions^20^ and a presentation session was also held. In order to adapt the sessions, a plan was devised for each session, including the identification of the competences to be stimulated, instructions for implementing the tasks and also the material needed for the sessions, suitably adapted for use in the Portuguese reality. For example, Portuguese proverbs were used for the executive function tests and for the memory stimulation the material was organized by using relevant episodes of Portuguese history (such as the 25º of April 1974) or relevant figures such as Amália Rodrigues etc. For the structured sessions, each was previously planned in detail. The sessions were conducted in group format and directed at autobiographical memory, visual and verbal memory, attention, language, abstract reasoning and executive functioning. On a daily basis, a reality orientation moment was implemented with a reality orientation board on which the day, date, current events etc. were listed. Further informal cognitive stimulation sessions were held twice a week. These sessions were held with the purpose of promoting communication and socialization among the group, valuing the elderly persons' competences, skills and willingness while also promoting concentration, memory and executive functions. These sessions were also group-based but were less structured and none of the sessions targeted a specific cognitive dimension, representing an opportunity for the participants to be engaged in a range of activities and discussions.^21^ The dynamic was also significantly different. There was a previous pool of prepared tasks (word games, fluency tasks, themes for discussion, news, discussion of special dates and events etc.) that were used according to the group adhesion. Movement sessions were also implemented twice a week following these periods.

As can be seen, some of the same approaches are used in mild to moderate Alzheimer patients, such as the Reality Orientation.^22^ Although there is no precise distinction between normal and pathological aging, particularly in illiterates and individuals with low educational levels who exhibit low levels of performance on psychological tests and neuropsychological measures,^8^ all the proposed tasks were considered to be stimulating for the participants.

Prior to the implementation of the program, there was an assessment session comprising a global measure of cognitive functioning, adapted and validated for Portuguese (including space and time orientation, immediate and delayed verbal recall, mental calculation, language, constructional ability and total score),^23^ and an information task (remote memory) was also included. Space and time orientation included questions such as "what day is it today?", "where are we now?". Verbal memory consisted of the presentation of three words that were repeated immediately after the presentation (immediate recall), or after a delay (delayed recall). Attention and calculus were evaluated by giving a number to the participant and asking them to subtract "3" from the given number until asked to stop. Language evaluation comprised oral and written tasks, using tasks for naming, repetition, understanding simple orders, reading and writing (sentence). Constructional abilities were evaluated by asking the participants to copy a drawing.

After completion of the program the evaluation was repeated.

## RESULTS

**General results.** Basic descriptive statistics were computed for baseline and follow up.

When comparing means between the two evaluation time-points, no significant differences were found in any of the variables considered (Wilcoxon Sign Rank tests, p>0.05).

Another notable aspect was the impact on non-cognitive dimensions. This was a significantly time-consuming project that required changes in the usual functioning and planning. It was felt by the people working with the elderly that they were "more engaged", "more present" and "more evolved" in the sequence of the program. It would have been of interest to ascertain whether the relatives also felt changes in daily living. Unfortunately, the majority of the sample lived alone and efforts to involve relatives, particularly sons and daughters, proved unsuccessful. Although not initially included, a posterior questionnaire based on Yishay and Daniels-Zide Rating Schema for acceptance was devised.^24^ It started with "Since these activities have been implemented at the day care center do you feel: Calmer (cessation of mourning); More satisfied (Morale), Better able to enjoy small daily things (capacity for enjoyment), better about yourself (restored self-esteem), more capable of doing your daily activities (Satisfaction with accomplishments). Responses were rated on five levels (much more, significantly more, same, worse, and much worse). This questionnaire was applied after the end of the program, excluding dropouts from the original sample and after implementation of the other activities, and was based on adaption of a brain lesion intervention in an aging context with no validation. Considering cessation of mourning, seven participants felt much calmer, two participants felt significantly calmer, and one participant perceived no difference. Regarding morale, seven participants felt much more satisfied, and three felt significantly more satisfied. The results were the same regarding capacity for enjoyment. Regarding self-esteem, three participants felt much better about this, five participants felt significantly better and two participants felt no difference. Regarding capacity to do daily activities, six participants felt much more capable and four felt significantly more capable. Generally speaking, the results were very significant (the vast majority scored "much more" on the questions, followed by "significantly more") and provided an indication of the positive evaluation by the elderly and the impact of these interventions at a non-cognitive level. However, it is interesting to note that results regarding self-esteem were less significant.

## DISCUSSION

Two lines of discussion can be developed. One regarding the impact of the intervention on cognitive status, and another regarding non-cognitive gains.

Regarding the impact of the intervention on cognitive status, our results showed that when comparing the neuropsychological measures assessed prior to and after implementation of the program, there was no difference in any of the variables considered.

We can interpret our results in a perspective of "Gains as losses of a lesser magnitude occurring later".^25^ As previously mentioned a study^14^ found that years of education were correlated with cognitive performance but not with cognitive decline, supporting the hypothesis of passive cognitive reserve with age. However, the authors regarded the fact they had included a very small subset of individuals with fewer than nine years of education as a limitation. In other words, according to the authors the lack of relationship between education and rate of cognitive decline was found in a relatively well-educated sample.^14^ On the other hand, other results suggest that age has a far more significant effect on rate of cognitive deterioration over a short period of time in persons with low education,^26^ strengthening our interpretation of gains as losses of a lesser magnitude occurring later.

This interpretation can of course be questioned. This study has several limitations including at the methodological level, some of which will be addressed in future studies. The most severe limitation was lack of a control group and therefore not having an alternative activity. This is the result of both the conditions in which the program was implemented on one hand, and its philosophy on the other. In terms of its facilities, the elderly day care center comprises two living rooms, one is a dining room and the other an activity room. Facility constraints made it difficult to accommodate experimental groups since all the elderly were present in the activity room at the time the group sessions were taking place. In terms of the philosophy of the intervention, given its context, eligible elderly could not be excluded. This was not an individual neuropsychological rehabilitation program. This meant that all attendees of the elderly day care center wishing to do so were actively enrolled in the activities, although some were excluded from the analysis as previously stated.

The approach employed with only two evaluation time points is also questionable. Although this can provide an estimate of rate of change, or detect no change in the present case, actual change in function between only two time points are difficult to detect, particularly with such a small sample.^27^

Nevertheless, there are novel aspects in this study that are worth reporting. One of the most relevant is the setting of the study, in which structured interventions are still relatively scarce. As noted by Fratiglioni et al.,^28^ cognitive training interventions have usually been done under laboratory or small-scale clinical conditions, showing that cognitive training helps normal elderly individuals do better on the specific task for which they were trained as compared with untrained individuals. However, it has been harder to prove that the improvement on any of the domains can be transferred to real world situations. Interventions at the community level are vital in establishing this transfer and remain scarce and difficult to implement.

The other relevant factor is the homogeneity of the sample, taking into consideration not only education, but also SES, income, social network, occupational attainment, living conditions, etc. In fact, the sample was remarkably similar in terms of its cognitive reserve, due to socio-historical conditions that are not easily repeatable.

In the review cited above,^28^ the hypothesis that both social network and leisure activity are implicated in the development of dementia and Alzheimer's disease (DA) was explored. The authors found that, when assessing the published longitudinal studies of lifestyles and cognition and dementia, that only a few included other indicators of socioeconomic status that can have a confounding role. This is reinforced by evidence^29^ that occupation can add to the effect of education. The authors proposed that mental stimulation after the educational years, such as professional occupations requiring high intellectual ability and few routine tasks, may result in a higher cognitive reserve capacity, pointing to the need to assess educational levels and professional occupations when evaluating neuropsychological abilities and particularly in dementia screening, underscoring the importance of socioeconomic variables.

According to Scarmeas and Stern (2003),^30^ exposure to an enriched environment, defined as a combination of more opportunities for physical activity, learning and social interaction, conditions we tried to implement in the present program, promotes not only a host of structural and functional changes in the brain but also influences the rate of neurogenesis. Richards et al. (2003)^28^ reinforced that the period when this stimulation can have impact is not limited to early stages of life. Fratiglione et al. (2004)^28^ reported results showing that the sensitive period extend at least until later life, reinforcing that not only cognitively stimulating activities, but also challenging leisure-time activities,^31^ can have an impact on cognitive maintenance.

On the other hand, considering other gains, two points can be made. Regarding the impact felt by the staff and the technicians, it should be emphasized that the program was very well received and it was reported that people were more "participative", "active" and "engaged". Since we tried to ensure this project involved everyone, there is of course the risk of being both judge and jury. Nevertheless, we are relatively confident of the validity of this judgment since there was additional work, or at least additional effort, involved.

Regarding the evaluation done by the elderly participants, as shown in the results, the vast majority scored "much more" followed by "significantly more" on the questions. This gives an indication that the elderly felt the program was useful and had an impact at a non-cognitive level. It is however interesting to note that results regarding self-esteem were less marked, probably reflecting the deep difficulty in adjusting to a society where elderly are less valued, a situation requiring more targeted approaches to reverse.^32^

In conclusion, as outlined previously, it will be of great significance if, within the community, simple and accessible programs could be implemented impacting cognitive status as well as other dimensions relevant to the lives of the elderly.

Our results suggest that programs specifically devised to be implemented in the community can have an impact in promoting the maintenance of cognitive status as well as in improving other non-cognitive aspects.

This is likely of great importance for those with low education that have a life of low stimulation and that, in old age, and according to the cognitive reserve concept,^10,12^ are less equipped to deal with losses, even those resulting from physiological aging. This investment, even in old age, is reinforced by previous work showing that an engaged lifestyle and cognitive function mutually influence each other in middle and old age non-demented persons, concluding that this reciprocal association may create a self-reinforcing, beneficial or adverse life course in middle and old age and discussing a "broader engagement hypothesis".^33^

Nevertheless, like all authors and as previously noted, we remain cautious when considering the impact of the stimulation program on rate of cognitive decline. There are several methodological issues that have to be addressed. Working in the community requires compliance with specific legal norms, logistic and ethical constraints as well as personal and social interactions etc. and above all, with the old person's real context of living.

Knowing the right activities, the right time and the right interaction of controllable factors for cognitive maintenance and Alzheimer prevention remain a mirage, particularly when education with its non-linear interactions is considered. Nevertheless, in the light of recent data regarding the prevalence of dementia showing that "It was estimated that 35.6 million people lived with dementia worldwide in 2010, with numbers expected to almost double every 20 years, to 65.7 million in 2030 and 115.4 million in 2050",^34^ we have no doubt that working at a community level will be of essence when developing interventions aimed at preventing dementia.

## Figures and Tables

**Table 1:** Mean results of performance at baseline and follow up.

	Prior to program implementation Mean result and standard Deviation	After program completion Mean result and standard Deviation
Orientation	8.64±1.336	9.14±1.167
Retention (immediate recall)	3.0±0.0	2.93±0.267
Attention and Calculus	3.07±2.2	2.86±1.99
Evocation (delayed recall)	2.07±1.072	2.29±0.825
Language	6.79±1.31	7.21±0.0975
Constructional Abilities	0.43±0.514	0.43±0.514
Total Cognitive Score	24.00±4.992	24.87±3.93
Information (remote memory)	10.86±1.29	10.36±3.296
